# Annexin A2 Regulates AKT Upon H_2_O_2_-Dependent Signaling Activation in Cancer Cells

**DOI:** 10.3390/cancers11040492

**Published:** 2019-04-07

**Authors:** Stéphanie Anais Castaldo, Tom Ajime, Gisela Serrão, Fábio Anastácio, Joana Teixeira Rosa, Carman Anthony Giacomantonio, Alison Howarth, Richard Hill, Patrícia Alexandra Madureira

**Affiliations:** 1 Centre for Biomedical Research (CBMR), Campus of Gambelas, University of Algarve, Building 8, Room 2.22, 8005-139 Faro, Portugal; stephanie.castaldo@cme.vib-kuleuven.be (S.A.C.); tommysawyer2002@gmail.com (T.A.); giselaserrao@gmail.com (G.S.); fabioanastacio@live.com.pt (F.A.); joana.t.rosa@gmail.com (J.T.R.); richard.hill@port.ac.uk (R.H.); 2 Department of Medicine, Dalhousie University, Halifax, Nova Scotia B3H 4R2, Canada; carman.giacomantonio@dal.ca; 3 Brain Tumour Research Centre of Excellence, Institute of Biomedical and Biomolecular Sciences, University of Portsmouth, PO1 2DT Portsmouth, UK; alison.howarth@port.ac.uk

**Keywords:** ANXA2, PTEN, PI3K/AKT, PRDX2, H_2_O_2_-dependent signaling

## Abstract

Hydrogen peroxide (H_2_O_2_) is a main second messenger in oncogenic signaling networks including the Ras and the growth factor receptor pathways. This is achieved predominantly through the oxidation of redox-sensitive cysteine (Cys) residues in proteins resulting in changes to their structure and function. We previously identified annexin A2 (ANXA2) as a redox regulatory protein that plays an important cellular role during oxidative stress and also promoting tumorigenesis. Here we investigated the role of ANXA2 in the regulation of H_2_O_2_-dependent signaling that drives tumor progression. We show that depletion of ANXA2 leads to the enhanced activation of AKT following either EGF/EGFR stimulation or oncogenic Ras transformation. The phosphatase and tensin homologue (PTEN) protein negatively regulates the PI3K/AKT pathway. We demonstrate that ANXA2 via its reactive Cys-8 residue, binds to PTEN and that the co-expression of PTEN and ANXA2, but not ANXA2 Cys-8-Ala mutant, inhibits AKT phosphorylation on Ser 473. These results indicate that ANXA2 is important for PTEN regulation within the PI3K/AKT signaling cascade. Furthermore, we also reveal that ANXA2 inversely regulates the expression of the peroxidase, peroxiredoxin 2, in a reactive oxygen species dependent manner.

## 1. Introduction

Hydrogen peroxide (H_2_O_2_) plays a key role as a second messenger in cell signal transduction, regulating cell proliferation, differentiation, migration and apoptosis [1,2,3]. This occurs primarily through the oxidation of redox-sensitive (reactive) cysteine (Cys) residues within proteins by H_2_O_2_ which results in changes to their structure and function [4,5,6].

The epidermal growth factor receptor (EGFR) signaling is commonly up-regulated in cancer, constituting an important pathway that regulates cell growth, survival, proliferation and differentiation [7,8]. EGFR signaling is initiated by growth factor (e.g., EGF) binding which stimulates dimerization and autophosphorylation of the receptor and subsequent downstream signaling by phosphorylation/activation of tyrosine kinases and the concomitant inhibition of protein tyrosine phosphatases and the phosphatase and tensin homologue (PTEN) protein. Particularly, the activation of the EGFR/PI3K/AKT pathway enables Rac to bind to and activate NADPH oxidase (NOX), ultimately leading to the production of H_2_O_2_ [9,10]. H_2_O_2_ reversibly oxidizes and inactivates the main inhibitor of the PI3K/AKT pathway, PTEN, allowing signal transduction to occur.

Another prominent H_2_O_2_-dependent signaling network associated with tumorigenesis is the Ras pathway. Ras is a small G-coupled protein, whose activity is regulated by association with GTP (promoted by Guanine Exchange Factors) generating an ON/active protein or GDP (promoted by its own GTPase activity) constituting the OFF/inactive Ras. The constitutive activation of Ras has been shown in approximately 30% of all cancers [11]. This occurs via mutation(s) of critical amino acid residue(s) leading to the loss of Ras GTPase activity [12]. Ras regulates three critical signaling networks, namely the Raf/MEK/ERK, PI3K/AKT and Ral-GDS pathways [12]. Ras/PI3K/AKT signaling induces ROS production through the activation of the NOX regulatory subunit, Rac [13].

To balance the proliferative and survival advantage of moderate reactive oxygen species (ROS) levels versus their damaging effect at high concentrations, cancer cells undergo a redox adaptation process through the induction of the antioxidant response [14]. We identified a novel redox regulatory protein, annexin A2 (ANXA2) that plays a key role in protecting cells from ROS induced death, supporting both tumor growth and chemo-resistance [1,4,15]. ANXA2 can exist in the cells as a monomer or a heterotetramer, where two ANXA2 molecules are bound together by a dimer of the protein, S100A10 [16,17]. We previously demonstrated that the redox regulatory function of ANXA2 is independent of S100A10 [1,14,15].

Here we show that ANXA2 depleted cells have enhanced phosphorylation of AKT upon EGF/EGFR activation or transformation with oncogenic H-RasV12. We demonstrate that ANXA2, via its reactive Cys-8 residue, can bind to the negative regulator of the PI3K/AKT signaling pathway, PTEN. Our co-expression studies reveal that ANXA2 positively regulates PTEN activity, leading to the inhibition of AKT. We also report for the first time that ANXA2 inversely regulates the peroxidase, PRDX2 in a reactive oxygen species (ROS) dependent manner.

## 2. Results

### 2.1. ANXA2 Depleted Cells Show Enhanced AKT Activation Upon EGF/EGFR Stimulation

**Figure 1:**
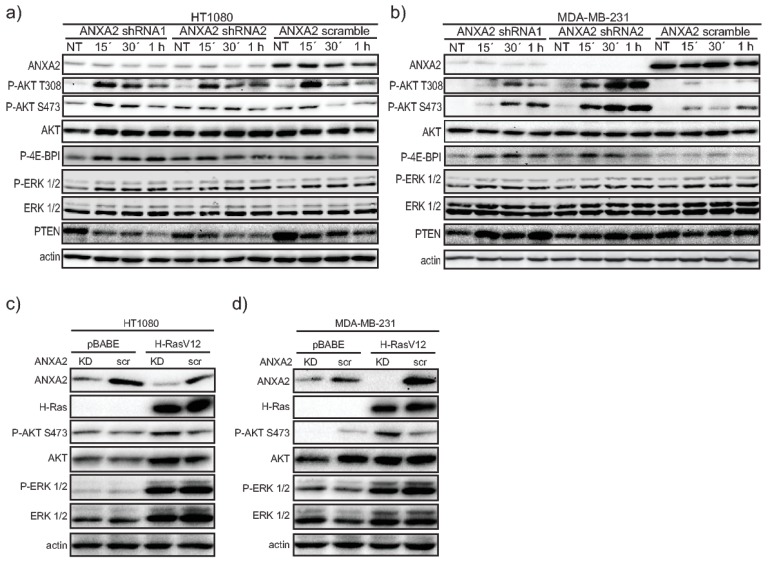
ANXA2 depleted cancer cells show enhanced AKT phosphorylation/activation upon H_2_O_2_ dependent signaling compared to control cells. (**a**) HT1080 or (**b**) MDA-MB-231 cancer cells depleted of ANXA2 (ANXA2 shRNA1, ANXA2 shRNA2) or controls (ANXA2 scramble) were serum starved for 6 h (HT1080) or for 16 h (MDA-MB-231) and either not treated (NT) or treated with 15 nM EGF for the times indicated. (**c**) HT1080 or (**d**) MDA-MB-231 ANXA2 knockdown (KD) and control (scr) cells expressing H-RasV12 or the empty vector pBABE were grown in serum free media for 6 h or 16 h, respectively, after what cells were lysed and 20 µg of each protein extract was subjected to SDS-PAGE, transferred onto nitrocellulose membranes and analyzed by western blotting with the antibodies indicated. Results are representative of at least three independent experiments (*n* ≥ 3).

To investigate the role of ANXA2 in the regulation of EGF/EGFR signaling, we established cancer cell lines depleted of ANXA2 (A549, HT1080 and MDA-MB-231 ANXA2 knockdown (KD)) and respective controls (ANXA2 scramble). The treatment of ANXA2 KD cells with 15 nM EGF over time led to a significantly higher activation of the PI3K/AKT signaling pathway, as assessed by enhanced AKT phosphorylation at Thr308 and Ser473, and increased activation of the PI3K/AKT target protein, P-4E-BPI in ANXA2 KD cells compared to their matched controls (Figure 1A,B and Supplementary Materials Figure S1). This result was concomitant with increased proliferation of ANXA2 depleted cells compared to their respective control cells following EGF treatment (Figure S2). We noted no significant difference in the activation of the MAPK/ERK 1/2 signaling pathway, evaluated through the phosphorylation of ERK 1/2, or PTEN expression in ANXA2 KD versus matched control cells (Figure 1A,B and Supplementary Materials Figure S1).

### 2.2. ANXA2 Depleted Cells Show Enhanced AKT Activation Upon Oncogenic Ras Transformation

We next questioned if ANXA2 could play a role in the regulation of other oncogenic H_2_O_2_ inducing signaling pathways. We transformed HT1080 and MDA-MB-231 ANXA2 depleted and control cells with oncogenic H-RasV12 or empty vector (pBABE). Cells depleted of ANXA2 and over-expressing oncogenic H-RasV12 showed enhanced pSer473 AKT (Figure 1C,D) compared to ANXA2 scramble cells over-expressing H-RasV12 or pBABE expressing cells. The activation of the MAPK/ERK1/2 signaling pathway was significantly higher in H-RasV12 expressing cells compared to controls (pBABE), however no differences were observed between ANXA2 depleted and matched control cells (Figure 1C,D).

### 2.3. ANXA2 Via Cys-8 Residue Interacts with PTEN and Regulates Its Activity

**Figure 2:**
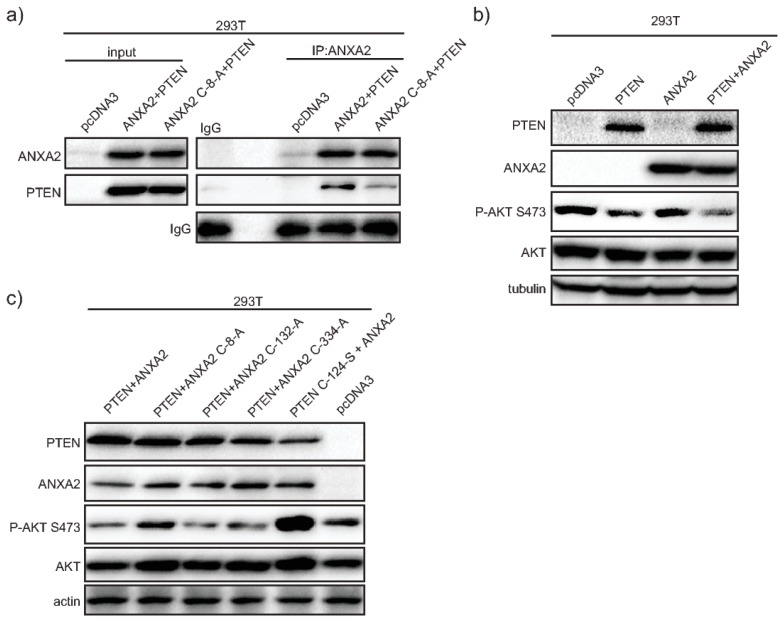
ANXA2 via its Cys-8 REDOX sensitive residue binds to PTEN and regulates its activity. (**a**) 293T cells transfected with either empty vector; ANXA2 and PTEN-GFP expression plasmids or ANXA2 Cys-8-Ala and PTEN-GFP expression plasmids were grown in 60 mm plates. Cells were washed with PBS, lysed with Triton X-100 lysis buffer degassed for 1 h on ice. 300 µg of each cell lysate was incubated with the indicated antibodies for 1 h at 4 °C and then 50 µL of pre-washed 50% slurry protein G-Sepharose™ (GE Healthcare) was added for 1 h at 4 °C. Beads were washed four times with 300 μL of lysis buffer, resuspended with 20 μL 2× SDS-PAGE loading buffer, boiled for 10 min, subjected to SDS-PAGE and analyzed by western blotting; 293T cells over-expressing (**b**) (1) empty vector, (2) PTEN, (3) ANXA2 or (4) ANXA2 and PTEN; or (**c**) (1) ANXA2 and PTEN, (2) ANXA2 Cys-8-Ala and PTEN, (3) ANXA2 Cys-132-Ala and PTEN, (4) ANXA2 Cys-334-Ala and PTEN, (5) ANXA2 and PTEN Cys-124-Ser or (6) pcDNA3 (empty vector) were grown in 60 mm plates in DMEM complete medium for 24 h. Cell lysates were prepared and 20 µg of each protein extract was subjected to SDS-PAGE, transferred onto nitrocellulose membranes and analyzed by western blotting with the antibodies indicated.

We observed enhanced activation of AKT in ANXA2 depleted compared to control cells in response to H_2_O_2_-dependent signaling. Based on these data we questioned if ANXA2 could interact with PTEN, the main inhibitor of the PI3K/AKT pathway to indirectly regulate AKT activity. To investigate this hypothesis we performed co-immunoprecipitation (Co-IP) assays using 293T cells over-expressing WT or mutant ANXA2 Cys-8-Ala (ANXA2^C8A^) and PTEN. These results showed that ANXA2 and PTEN interact via the reactive Cys-8 residue of ANXA2, since mutation of this residue to alanine abolished the ANXA2/PTEN complex (Figure 2A).

To further investigate if and how ANXA2 regulated PTEN activity, we performed transient transfections using 293T cells, which express very low endogenous levels of ANXA2 and PTEN, with a combination of plasmids expressing either WT or mutant ANXA2 and/or PTEN proteins (Figure 2B,C). These data demonstrated that expression of PTEN alone led to a slight decrease in AKT activity compared to control cells. However, AKT inhibition was more striking in cells over-expressing PTEN and ANXA2 (Figure 2B). Interestingly, expression of ANXA2 in 293T cells did not induce any changes in pSer473 AKT levels compared to control cells (293T pcDNA3) (Figure 2B). ANXA2 contains three cysteine (Cys) residues. To determine which ANXA2 Cys residue(s) were important for PTEN activity regulation we co-expressed PTEN with each mutant ANXA2 and evaluated the level of pSer473 AKT. The expression of ANXA2, ANXA2 Cys-132-Ala (ANXA2^C132A^) or ANXA2 Cys-334-Ala (ANXA2^C334A^) mutant proteins in 293 T cells over-expressing PTEN resulted in the inhibition of AKT, measured by reduced pSer473 AKT compared to control 293T cells transfected with empty pcDNA3 vector (Figure 2C). However, co-expression of ANXA2^C8A^ and PTEN did not inhibit pSer473 AKT levels compared to control cells (Figure 2C). Taken together these data indicate that the Cys-8 residue of ANXA2 plays an important role in maintaining PTEN activity. Interestingly, we observed that over-expression of the PTEN^C124S^ mutant enhanced pSer473 AKT compared to control cells. These results suggest that PTEN ^C124S^ might function as a dominant negative protein, having an opposite effect on AKT regulation compared to its WT counterpart protein.

### 2.4. ANXA2 Regulates PRDX2 Expression

We noted that the same ANXA2 shRNAs led to more efficient knockdown of ANXA2 in MDA-MB-231 cells compared to HT1080 or A549 cell lines (Supplementary Materials Figure S3). Since ANXA2 is a H_2_O_2_ scavenger we investigated the ROS levels in these cells using 2′,7′-dichlorofluorescin diacetate (DCF-DA) reagent. We observed higher levels of ROS in HT1080 and A549 ANXA2 KD cells compared to their respective controls; interestingly, this was not observed in the MDA-MB-231 isogenic pair (Supplementary Materials Figure S4). These data suggested that a more efficient depletion of ANXA2 in the HT1080 or A549 cells likely resulted in a survival and/or proliferation disadvantage due to excess ROS.

**Figure 3:**
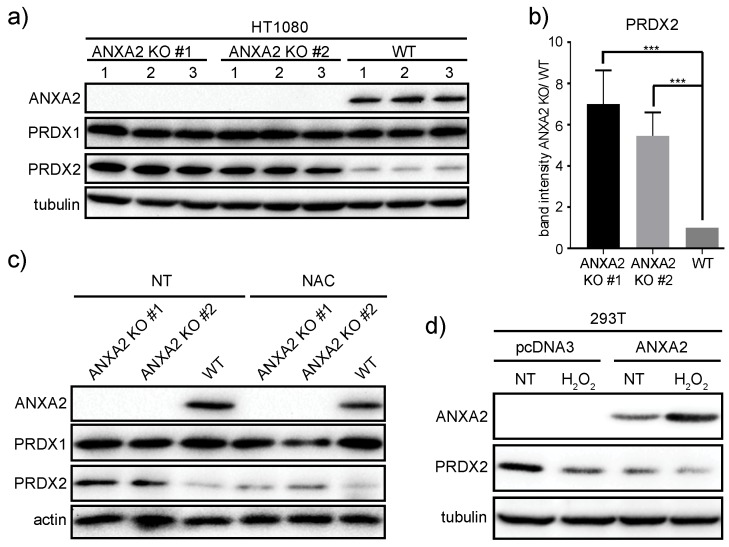
ANXA2 regulates PRDX2 expression in a ROS dependent way. (**a**) HT1080 ANXA2 knockout or WT cells were grown in complete media until 80% confluency. After what cells were lysed and 20 µg of each protein extract was subjected to SDS-PAGE, transferred onto nitrocellulose membranes and analyzed by western blotting with the antibodies indicated. Cells were loaded in triplicates; (**b**) Quantification analysis of PRDX2 immunoblots from (**a**). Quantification was performed using Image J software. Tubulin immunoblot was used as a loading control for normalization of the quantification. Error bars represent Standard Deviations. Statistical analysis was evaluated using two-tailed Student’s *t*-test. In every case a *p*-value of less than 0.05 (*), less than 0.01(**) and 0.001 (***) was considered statistically significant; (**c**) HT1080 ANXA2 knockout sub-populations (ANXA2 KO #1; ANXA2 KO #2) or wild-type (WT) cells were grown in complete media either not supplemented (left panel) or supplemented with 5 mM NAC (right panel) for 48 h. After what cells were lysed and 20 µg of each protein extract was subjected to SDS-PAGE, transferred onto nitrocellulose membranes and analyzed by western blotting with the antibodies indicated; (**d**) 293T cells were transiently transfected with either pcDNA3 (empty) vector or pcDNA3-ANXA2 plasmids as indicated in the figure. 24 h after transfection these cells were either not treated or treated with 100 µM of H_2_O_2_ for 24 h. Cells were then lysed and 20 µg of each protein extract was subjected to SDS-PAGE, transferred onto nitrocellulose membranes and analyzed by western blotting with the antibodies indicated. Results are representative of three independent experiments (*n* = 3).

We next investigated the expression of the H_2_O_2_ scavengers, PRDX1 and PRDX2, in our isogenic cell lines (Supplementary Materials Figure S5). We observed that ANXA2 depletion led to the upregulation of PRDX2 in MDA-MB-231 cells compared to control cells (Supplementary Materials Figure S5B), but this was not observed in HT1080 ANXA2 KD versus control cells (Supplementary Materials Figure S5A). Of note, we were not able to detect PRDX2 in A549 cells (confirmed in Supplementary Materials Figure S5A). We observed that cells depleted of ANXA2 and over-expressing oncogenic H-RasV12 showed enhanced expression of PRDX2 compared to ANXA2 scramble cells over-expressing H-RasV12 or pBABE expressing cells (Supplementary Materials Figure S5D,E). To investigate if a more efficient depletion of ANXA2 in HT1080 cells could lead to increased expression of PRDX2, we abrogated the *ANXA2* gene in the HT1080 cells by CRISPR/Cas9. We noted an approximately 5-6-fold increase in PRDX2 (but not PRDX1) expression in these cells compared to HT1080 WT cells (Figure 3A,B and Supplementary Materials Figure S6A). We also established MDA-MB-231 *ANXA2* KO cells and observed the up-regulation of PRDX2 compared to control cells (Supplementary Materials Figure S6B). Importantly, cells which reverted to the WT phenotype by non-homologous recombination maintained similar levels of PRDX2 compared to MDA-MB-231 WT cells (Supplementary Materials Figure S6B, lanes 2,5,6). In contrast, depletion of PRDX2 by shRNA did not lead to up-regulation of ANXA2 in either MDA-MB-231, MCF7 or HCT-116 cancer cells (Supplementary Materials Figure S7A–C).To investigate if the up-regulation of PRDX2 in HT1080 *ANXA2* KO cells was regulated by ROS, we treated these cells with the antioxidant N-acetyl cysteine (NAC). We observed the down-regulation of PRDX2 upon NAC incubation, indicative that regulation of PRDX2 was ROS dependent (Figure 3C).

We next over-expressed ANXA2 in 293T cells and observed the down-regulation of PRDX2 compared to control cells transfected with pcDNA3 (Figure 3D). Interestingly, while a 24h exposure with 100 µM H_2_O_2_ led to up-regulation of ANXA2, this oxidative stress induced the down-regulation of PRDX2.

**Figure 4:**
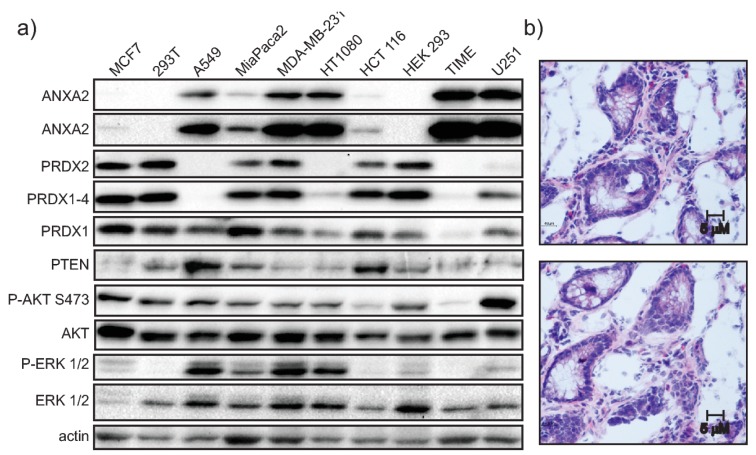

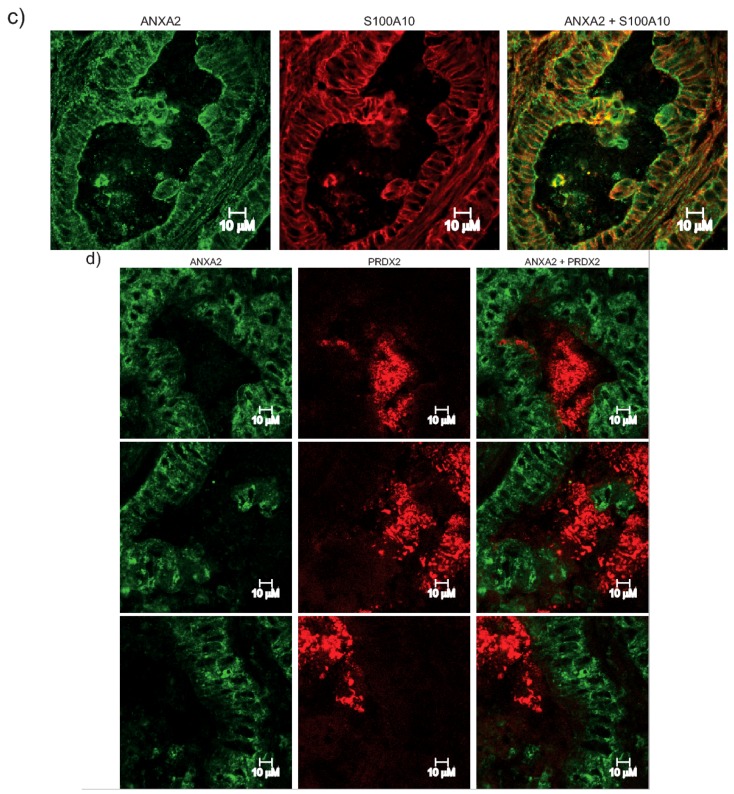
Western blot analysis of a panel of cell lines and clinical samples. (**a**) MCF7, 293T, A549, MiaPaca2, MDA-MB-231, HT1080, HCT116, HEK 293, TIME or U251 cells were lysed and 20 µg of each protein extract was subjected to SDS-PAGE, transferred onto nitrocellulose membranes and analyzed by western blotting with the antibodies indicated. This is a representative experiment of *n* > 3; Colon tumor clinical samples were flash frozen and sectioned using a cryostat. (**b**) Sections were then H&E stained or (**c**,**d**) fixed with methanol and immunostained with the antibodies indicated, followed by immunofluorescence staining with secondary antibodies (ANXA2-green; S100A10-red; PRDX2-red).

We further assessed the expression levels of ANXA2 and PRDX2 in a panel of cell lines by western blotting (Figure 4A). This analysis showed that cells expressing high levels of ANXA2 have low levels of PRDX2 and vice-versa, in agreement with the data described above. Cell signaling analysis showed that the activation of the PI3K/AKT and MAPK/ERK 1/2 pathways was variable for each cell line and did not show a clear correlation with the expression levels of either ANXA2, PRDX1-4 or PTEN.

We analyzed the distribution/localization of ANXA2 and PRDX2 in patient colon cancer tissue sections (Figure 4B,D and Supplementary Materials Figure S8A). We noted that regions where the expression of ANXA2 was high showed low to no expression of PRDX2 and vice-versa. Dual staining for the nuclear marker hnRNP A2/B1 and ANXA2 showed the presence of cells in regions that have low or no expression of ANXA2 (Supplementary Materials Figure S8B). Double staining for ANXA2 and S100A10 (ANXA2 binding partner [17]), showed co-localization of these proteins as predicted (Figure 4C and Supplementary Materials Figure S8C).

**Figure 5:**
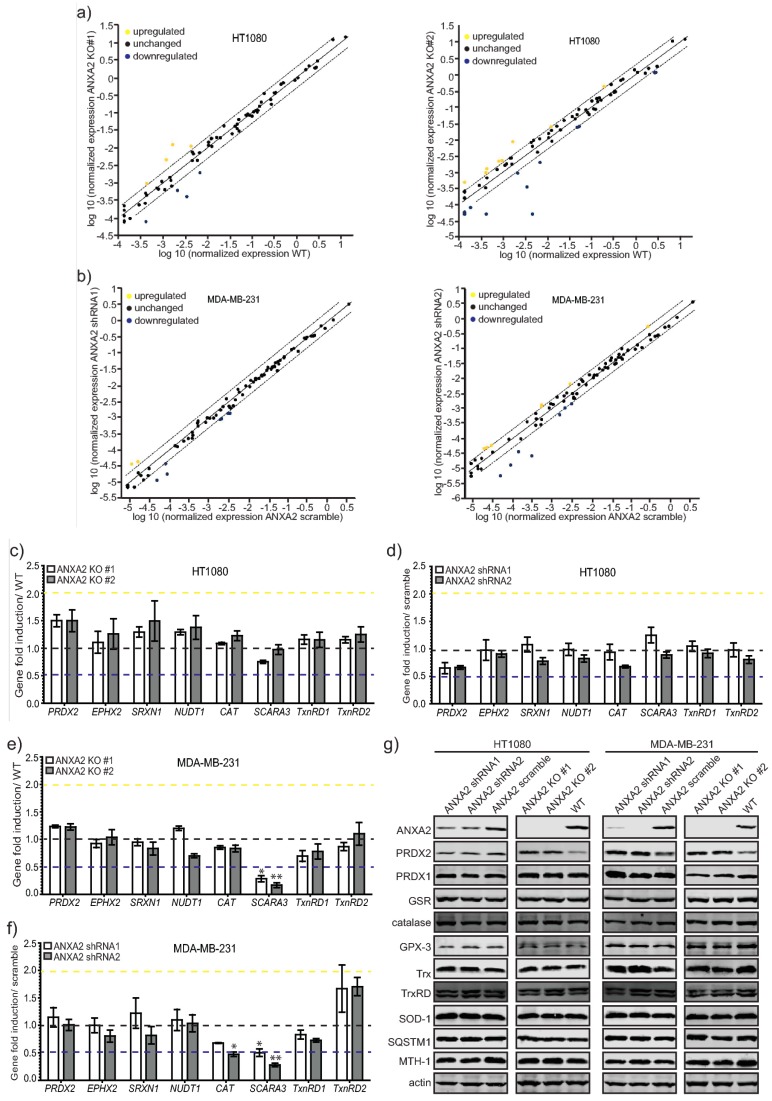
Analysis of ROS related genes and proteins in ANXA2 depleted versus control cancer cells. (**a**) HT1080 ANXA2 KO #1; ANXA2 KO #2 or WT or (**b**) MDA-MB-231 ANXA2 shRNA1; ANXA2 shRNA2 or ANXA2 scramble cells were plated in 100 mm plates for 48 h. After what RNA extraction was performed using the RNeasy mini kit (Qiagen, Manchester, UK) according to the manufacturer′s instructions. A panel of 86 ROS dependent genes was analysed using the RT^2^ Profiler™ PCR Array Human Oxidative Stress (Qiagen, Manchester, UK) according to the manufacturer’s instructions in a LightCycler 96 instrument (Roche, Basel, Switzerland). (**c**) HT1080 ANXA2 KO #1; ANXA2 KO #2 or WT; (**d**) HT1080 ANXA2 shRNA1; ANXA2 shRNA2 or ANXA2 scramble; (**e**) MDA-MB-231 ANXA2 KO #1; ANXA2 KO #2 or WT; (**f**) MDA-MB-231 ANXA2 shRNA1; ANXA2 shRNA2 or ANXA2 scramble cells were plated in 100 mm plates for 48 h. RNA extraction was performed using the RNeasy mini kit (Qiagen, Manchester, UK) according to the manufacturer´s instructions. The gene expression was determined by qRT-PCR using the One-step NZYSpeedy RT-qPCR Green kit (Nzytech, Lisbon, Portugal) according to manufacturer´s instructions. The reaction was carried out using the Lightcycler 96 instrument (Roche, Basel, Switzerland). Gene expression levels were normalized to RPLP0 mRNA using the 2^−ΔΔCT^ method (Livak KJ, Schmittgen TD). Error bars represent the standard deviation obtained from three independent experiments. Statistical analysis was evaluated using two-tailed Student’s *t*-test. In every case a *p*-value of less than 0.05 (*), less than 0.01(**) and 0.001 (***) was considered statistically significant. (**g**) HT1080 ANXA2 shRNA1, ANXA2 shRNA2 and scramble (first panel); HT1080 ANXA2 KO #1, ANXA2 KO #2 and WT (second panel); MDA-MB-231 ANXA2 shRNA1, ANXA2 shRNA2 and scramble (third panel); MDA-MB-231 ANXA2 KO #1, ANXA2 KO #2 and WT (fourth panel) cells were lysed and 20 µg of each protein extract was subjected to SDS-PAGE, transferred onto nitrocellulose membranes and analyzed by western blotting with the antibodies indicated. Results are representative of three independent experiments (*n* = 3).

We performed a more thorough analysis of ROS related genes in HT1080 *ANXA2* KO vs. WT and MDA-MB-231 ANXA2 KD vs scramble cell lines using the Human Oxidative Stress RT^2^ Profiler™ (Qiagen, Manchester, UK). The results depicted on Supplementary Materials Table S1 and Figure 5A,B did not show substantial differences in the expression of ROS related genes in the ANXA2 depleted versus control cells. We further validated the expression of a subset of these genes that showed modest differential expression changes in the ANXA2 depleted versus control cells by qRT-PCR (Figure 5C–F). We observed a 1.5 fold up-regulation of *PRDX2* in HT1080 and MDA-MB-231 ANXA2 KO compared to control cells (Figure 5C,E). We observed a 1.5–2 fold induction of *TrxRD2*, and down regulation of *SCARA-3* in MDA-MB-231 ANXA2 depleted compared to control cells (Figure 5E,F). We also investigated the expression of ROS related proteins. We did not observe significant differences in the expression of these proteins in ANXA2 depleted versus control cells, with the exception of PRDX2 (Figure 5G). Even though there were modest differences in the expression of *CATALASE* and *TrxRD2* genes in MDA-MB-231 ANXA2 KD compared to control cells we did not observe significant differences at the protein levels. Of note we could not detect SCARA-3 protein in our extracts.

## 3. Discussion

We previously identified ANXA2 as a redox regulatory protein that plays an important role during oxidative stress and tumorigenesis [1]. With this work, we show for the first time, that depletion of ANXA2 in cancer cells leads to enhanced activation of AKT in response to either EGF/EGFR activation or oncogenic H-RasV12 transformation (Figure 1 and Supplementary Materials Figure S1). The main inhibitor of the PI3K/AKT pathway, PTEN, contains reactive Cys residues in its catalytic domain that can be readily oxidized by H_2_O_2_ inactivating its phosphatase function [18]. Our co-immunoprecipitation studies revealed that ANXA2 interacts with PTEN via ANXA2 Cys-8 residue (Figure 2A). These data in conjunction with the decreased activation of AKT observed in ANXA2-containing compared to ANXA2 depleted cells upon H_2_O_2_-dependent signaling (Figure 1 and Supplementary Materials Figure S1) suggested that ANXA2 positively regulates PTEN activity. Co-transfection studies using a combination of WT or mutants ANXA2 and PTEN expression plasmids, confirmed that ANXA2 positively regulates PTEN inhibition of pSer473 AKT, in a Cys-8 residue of ANXA2 dependent way (Figure 2C,D). Importantly, expression of ANXA2 alone in 293T cells did not inhibit pSer473 AKT compared to 293T control cells (293T transfected with pcDNA3) (Figure 2B, lane 3). These data suggest that ANXA2 does not directly regulate AKT activity, but does so via PTEN.

In this work we show for the first time that ANXA2 regulates the expression of the peroxidase, PRDX2. In summary, we observed increased expression of PRDX2 upon depletion of ANXA2 in cancer cells (Figure 5G and Supplementary Materials Figure S5). Of note, depletion of ANXA2 by shRNA was insufficient to induce PRDX2 up-regulation in HT1080 ANXA2 KD cells or in MDA-MB-231 and HT1080 pBABE cells, whose down-regulation of ANXA2 was less efficient compared to MDA-MB-231 ANXA2 KD cells (Figures S3 and S5). However, *ANXA2* gene deletion in HT1080 and MDA-MB-231 cells led to the over-expression of PRDX2 compared to their respective WT cells (Figure 3, Figure 5 and Figure S6). Together these results suggest that a threshold for ANXA2 depletion is required to trigger PRDX2 up-regulation in cancer cells. We demonstrated that PRDX2 regulation by ANXA2 was ROS dependent (Figure 3C). In this sense, a pronounced down-regulation of ANXA2 might be necessary to induce sufficient accumulation of ROS leading to the subsequent up-regulation of PRDX2 to compensate for the REDOX imbalance. In fact, cells over-expressing H-RasV12, which leads to up-regulation of intracellular ROS, and depleted of ANXA2 showed a significantly higher expression of PRDX2 compared to cells expressing endogenous levels of ANXA2 and H-RasV12 (Supplementary Materials Figure S5). Analysis of intracellular ROS revealed that HT1080 and A549 ANXA2 KD cells have significantly higher levels of endogenous ROS compared to their respective controls, while this was not observed in MDA-MB-231 ANXA2 KD cells (Figure S4). We observed up-regulation of PRDX2 in the MDA-MB-231 ANXA2 KD compared to control cells, which did not occur in the isogenic HT1080 or A549 cells (Supplementary Materials Figure S4). Taken together these data support that up-regulation of PRDX2 in MDA-MB-231 ANXA2 KD cells leads to REDOX homeostasis. Importantly, over-expression of ANXA2 led to down-regulation of PRDX2 in 293T cells (Figure 3D), further supporting that ANXA2 inversely regulates the cellular expression of PRDX2. Interestingly, when we depleted PRDX2 in cancer cells we did not observe up-regulation of ANXA2 (Supplementary Materials Figure S7). Analysis of ANXA2 and PRDX2 protein expression in a panel of cell lines further showed an inverse relationship between ANXA2 and PRDX2 levels (Figure 4A). Furthermore, expression patterns of ANXA2 and PRDX2 in colon cancer sections revealed that the distribution of these two proteins is highly distinct. Areas with high ANXA2 expression display little to no PRDX2 and vice-versa (Figure 4D and Supplementary Materials Figure S8A). PRDX2 and ANXA2 share many characteristics, including similar cellular distribution, high cytoplasmic abundancy with a small fraction in the nucleus [19,20], calcium-dependent plasma membrane binding [17,21,22], lipid raft co-localization [20,23], ion channels regulation [17,24,25], both directly inactivate H_2_O_2_ and are recycled/reduced by the thioredoxin system [1,20] and both protect cellular DNA from oxidative damage [15,26]. However, it is highly likely that ANXA2 and PRDX2 also have distinct functions and interact with different proteins and targets within cells.

A study by Cao et al. found that PRDX1 binds to PTEN protecting it from oxidation-induced inactivation and indirectly regulating AKT phosphorylation upon Ras signaling [27]. The authors also show that PRDX2 was not able to bind to PTEN, which is in agreement with our results, demonstrating that PRDX2 up-regulation is not able to impede the enhanced AKT phosphorylation observed upon depletion of ANXA2 in cancer cells.

Over-oxidation of PRDXs has been linked to cell signaling through the “floodgate model,” in which PRDXs consume low levels of endogenous peroxides, but enhanced generation leads to over-oxidation/degradation and increase in H_2_O_2_ that acts as a second messenger [28]. Compared to PRDX2, ANXA2 seems to be more resistant to oxidation during H_2_O_2_-dependent signaling [1,4]. In fact, when we treated 293T cells with a high oxidative insult (100 µM of H_2_O_2_) we observed decreased levels of PRDX2, while ANXA2 expression increased (Figure 3D). In these experiments we transfected 293T cells with the ANXA2-pcDNA3 plasmid to transiently express ANXA2 in these cells. It has been previously shown that H_2_O_2_ induces the CMV promoter [29]. For this reason, it is likely that ANXA2 up-regulation in H_2_O_2_ treated cells is due at least in part to H_2_O_2_ induced CMV promoter activation. Nevertheless, Madureira et al. have previously shown that the treatment of ANXA2 low expressing cells, 293T and MCF7, with 100 μM H_2_O_2_ over a long period of time (two weeks) led to up-regulation of ANXA2 in these cells [1].

## 4. Materials and Methods

### 4.1. Cell Culture, Transfections and Cell Lines

MDA-MB-231, A549, MCF7, MiaPaca2, HCT 116, U251, HEK 293, HT1080 and 293T cell lines were obtained from ATCC and grown in DMEM (HyClone™, Pittsburgh, PA, USA) supplemented with 10% fetal bovine serum (FBS), 20 mM L-Glutamine and 100 U/mL penicillin/streptomycin. TIME cells were obtained from Dr McMahon and grown in EGM2 medium (Lonza, Basel, Switzerland) supplemented with 2% FBS and 100 U/mL of penicillin/streptomycin. Cells were maintained in a humidified incubator at 37 °C with 5% CO_2_. ANXA2 depleted cell lines and/or over-expressing H-RasV12 or pBABE were obtained by transfection of Phoenix cells with 4 μg of the plasmids described on table SII, using 8 μL of jetPRIME (Polyplus, Strasbourg, France) transfection reagent according to manufacturers’ instructions. 48 h after transfection the target cells were infected with Phoenix supernatants and selected with 2–5 μg/mL of puromycin for pSUPER-retro-puro or pBABE or 1 mg/mL neomycin for pSUPER-retro-neo backbone plasmids. HT1080 and MDA-MB-231 ANXA2 KO cell lines were obtained by tranfection of the WT cells with 3 µg of pANXA2-gRNA1-px459-V2 or pANXA2-gRNA2-px459-V2 using 6 µL of jetPRIME transfection reagent according to the manufacturers’ instructions. 48 h after transfection the cells were selected with 5 µg/mL of puromycin. Serial dilutions were performed to obtain HT1080 and MDA-MB-231 ANXA2 KO sub-populations. For transient transfection and co-immunoprecipitation experiments, 293T cells in 6 wells plates were transfected for 24–48 h with 1 μg of either pcDNA3, pcDNA3-ANXA2, pcDNA3-ANXA-Cys-8-Ala, pcDNA3-ANXA-Cys-132-Ala, pcDNA3-ANXA-Cys-334-Ala, pcDNA3.1-PTEN, pcDNA3.1-PTEN-Cys-124-Ser, or GFP-PTEN plasmids, described below, using 2 μL (single plasmid transfection) or 4 μL (co-transfection) of jetPRIME transfection reagent according to the manufacturers’ instructions.

### 4.2. Plasmids and Transfections

Construction of shRNA and CRISPR/Cas9 plasmids are described on Supplementary Materials Table S2. Construction of pcDNA3-ANXA2, pcDNA3-ANXA2-Cys-8-Ala, pcDNA3-ANXA2-Cys-132-Ala and pcDNA3-ANXA2-Cys-334-Ala plasmids is described in [1]. PTEN-Wt and PTEN-Cys-124-Ser cDNAs were cloned into pcDNA3.1 vector at restriction sites BamHI/NotI to generate pcDNA3.1-PTEN and pcDNA3.1-PTEN-Cys-124-Ser these plasmids were kindly provided by Dr. Wolfgang Link. GFP-PTEN was a gift from Dr. Alonzo Ross (Addgene plasmid # 13039). The plasmids pBabe-puro (1764) and pBabe-puro H-RasV12 (39526) were purchased from Addgene, Watertown, MA, USA.

ANXA2 depleted cell lines and/or over-expressing H-RasV12 or pBABE were obtained by transfection of Phoenix cells with 4 μg of the plasmids described on Supplementary Materials Table S2, using 8 μL of jetPRIME (Polyplus) transfection reagent according to manufacturers’ instructions. 48 h after transfection the target cells were infected with Phoenix supernatants and selected with 2–5 μg/mL of puromycin for pSUPER-retro-puro or pBABE or 1 mg/mL neomycin for pSUPER-retro-neo backbone plasmids. HT1080 and MDA-MB-231 ANXA2 KO cell lines were obtained by tranfection of the WT cells with 3 µg of pANXA2-gRNA1-px459-V2 or pANXA2-gRNA2-px459-V2 using 6 µL of jetPRIME transfection reagent according to the manufacturers’ instructions. 48 h after transfection the cells were selected with 5 µg/mL of puromycin. Serial dilutions were performed to obtain HT1080 and MDA-MB-231 ANXA2 KO sub-populations. For transient transfection and co-immunoprecipitation experiments, 293T cells in 6 wells plates were transfected for 24–48 h with 1 μg of either pcDNA3, pcDNA3-ANXA2, pcDNA3-ANXA-Cys-8-Ala, pcDNA3-ANXA-Cys-132-Ala, pcDNA3-ANXA-Cys-334-Ala, pcDNA3.1-PTEN, pcDNA3.1-PTEN-Cys-124-Ser, or GFP-PTEN plasmids, described below, using 2 μL (single plasmid transfection) or 4 μL (co-transfection) of jetPRIME transfection reagent according to the manufacturers’ instructions.

### 4.3. Antibodies

Antibodies are listed in Supplementary Materials Table S3.

### 4.4. Immunoprecipitation Assays

For co-immunoprecipitations, cells were washed with PBS, lysed with Triton X-100 lysis buffer (50 mM Tris pH 7.5, 0.2% Triton X-100, 15 mM NaCl, 2 mM EGTA, 1 mM EDTA, protease inhibitors, 5 mM NaVO_4_, 10 mM NaF) degassed for 1 h. Cell lysates were incubated with specific antibodies for 1 h at 4 °C and then pre-washed 50% slurry protein G-Sepharose™ (GE Healthcare, Pittsburgh, PA, USA) was added for 1 h at 4 °C. Immunoprecipitates were washed four times with 300 μL of lysis buffer, resuspended with 20 μL 2× SDS-PAGE loading buffer, boiled for 10 min, subjected to SDS-PAGE and analyzed by western blotting.

### 4.5. Western Blotting

Cells were treated as described in the results section and extracts were prepared with lysis buffer (2 mM EGTA, 1 mM EDTA, 50 mM Tris pH 7.5, 120 mM NaCl, 0.1% Na deoxycholate, 1% NP-40, 10 mM NaF, 5 mM NaVO_4_, protease inhibitors). For western blotting, 20 μg of each cell lysate was subjected to SDS-PAGE, transferred onto a nitrocellulose membrane, incubated with appropriate antibodies and visualized using ChemiDoc™ XRS (BioRad^®^, Watford, UK).

### 4.6. qRT-PCR

RNA was isolated using the RNeasy Mini Kit (Qiagen) according to manufacturer’s instructions. The gene expression was measured by qRT-PCR using the One-step NZYSpeedy RT-qPCR Green kit (Nzytech, Lisbon, Portugal) according to manufacturer´s instructions in a Lightcycler 96 instrument (Roche, Basel, Switzerland). Gene expression levels were normalized to RPLP0 mRNA using the 2^−ΔΔCT^ method [30]. Error bars represent the standard deviation obtained from average of triplicate samples from three independent experiments. The primers used for qRT-PCR are on Supplementary Materials Table S4.

### 4.7. Immunofluorescence Studies

Snap frozen tumors were sectioned on a cryostat and mounted on slides. Slides were fixed for 30 min using −20 °C cold methanol. Slides were incubated in a humidified chamber at 37 °C for 30 min with swine serum (Dako, Glostrup, Denmark). The serum was removed and the slides were incubated with anti-ANXA2 (dil 1:500) and anti-PRDX2 (dil 1:500) for 1 h in a humidified chamber at 37 °C. After washing three times with PBS for 10 min at RT, slides were incubated with the appropriate FITC-conjugated secondary antibodies in a humidified chamber at 37 °C for 1 h. After washing as described above, chamber slides were mounted on glass cover slips using Vector mounting solution for fluorescence (Vector Laboratories, UK) and viewed using a Zeiss LSM 510 META -Laser Scanning Confocal Microscope (Carl Zeiss Inc., Oberkochen, Germany.

### 4.8. H&E Staining

Snap frozen tumors were sectioned using a cryostat and mounted on slides. Samples were incubated in 95% alcohol for 2 min and 70% alcohol for 2 min. Afterwards they were washed briefly in distilled water. Slides were immersed in Harris hematoxylin solution for 8 min and then washed in running tap water for 5 min. After washing, slides were incubated in 1% acid alcohol for 30 s and washed in running tap water for 1 min. Slides were incubated in 0.2% ammonia water for 45 s and washed again in running tap water for 5 min. Slides were rinsed with 95% alcohol and counterstained in eosin-phloxine B solution 1 min. Following incubation samples were dehydrated with 95% alcohol for 5 min and two changes of absolute alcohol for 5 min. Slides were cleared by 2 changes of xylene for 5 min each. Slides were mounted with xylene based mounting medium (DAKO, Glostrup, Denmark) and visualized using an optical microscope (Axiovert 40C, Carl Zeiss Inc., Oberkochen, Germany).

### 4.9. Clinical Samples Collection

Colon carcinoma samples were obtained from patients at the time of surgery. Patients´ consent was obtained and the experiments conformed to the principles set out in the WMA Declaration of Helsinki and the Department of Health and Human Services Belmont Report. Ethical approval was obtained for each procedure from the Research Ethics Board, (VG Hospital, Halifax, Nova Scotia, Canada; ethical code: CDHA-RS/2010-111, 17 September 2010).

### 4.10. Oxidative Stress RT2 PCR Profiler Array

1 × 10^6^ cells were grown in 100 mm plates for 24 h. After what RNA was extracted using the RNeasy Plus Mini Kit (Qiagen, Manchester, UK) according to the manufacturer´s instructions. RNA quality was analyzed using an RNA 6000 Nano Kit chip assay (Agilent, Santa Clara, CA, USA) in a 2100 Bioanalyzer (Agilent, Santa Clara, CA, USA) to ensure RNA extracts had an integrity index value greater than 9.0. Two µg of each RNA was used for cDNA synthesis using the RT2 first strand kit (Qiagen, Manchester, UK) according to the manufacturer’s instructions. The RT^2^ Profiler™ PCR Array Human Oxidative Stress; ref: PAHS-065Z (Qiagen, Manchester, UK) was performed according to the manufacturer’s instructions in a LightCycler 96 instrument (Roche, Basel, Switzerland).

### 4.11. Statistical Analysis

The statistical significance was evaluated using two-tailed Student’s *t*-test. The *p*-values were calculated from at least three independent experiments (*n* ≥ 3).

### 4.12. Supplementary Materials and Methods

Materials and Methods related to supplementary Figures are described in Appendix A.

## 5. Conclusions

**Figure 6:**
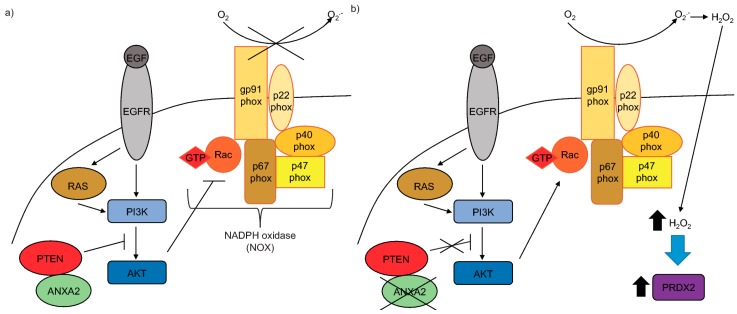
Proposed model for ANXA2 regulation of AKT activation. (**a**) ANXA2 binds to and positively regulates the activity of PTEN. This mechanism might play a role in the regulation of H_2_O_2_-dependent signaling, e.g., EGFR/EGF or RAS, leading to inactivation of the PI3K/AKT signaling pathway. (**b**) In the absence of ANXA2, PTEN activity is inhibited leading to enhanced AKT activation. The activation of the EGFR/PI3K/AKT or RAS/PI3K/AKT signaling pathways enable Rac to bind and activate NADPH oxidase (NOX), ultimately leading to the production of H_2_O_2_. Increase in intracellular H_2_O_2_ levels induce PRDX2 up-regulation.

Our data allowed us to propose the model depicted in Figure 6. In conclusion, we provide mechanistic evidence that ANXA2 via its Cys-8 residue has a key role in positively regulating PTEN to inhibit AKT phosphorylation at Ser473 activation site. The co-expression and co-immunoprecipitation studies in conjunction with our observation that ANXA2 depleted cells show enhanced phosphorylation of AKT following H_2_O_2_-dependent signaling compared to control cells support that direct regulation of PTEN by ANXA2 might constitute an important mechanism in regulating AKT phosphorylation following H_2_O_2_-dependent signaling (e.g., EGF/EGFR; Ras V12) (Figure 6A). However we cannot rule out the possibility that other molecular mechanisms might be involved in the regulation of AKT activity by ANXA2 during H_2_O_2_-dependent signaling. Activation of the EGF/PI3K/AKT or RAS/PI3K/AKT networks enable Rac to bind to and activate NOX. This ultimately drives the increase in intracellular H_2_O_2_ and potently induces the levels of the peroxiredoxin, PRDX2 to compensate for the REDOX unbalance and buffer/inactivate the excess H_2_O_2_ (Figure 6B).

## Supplementary Materials

The following are available online at https://www.mdpi.com/2072-6694/11/4/492/s1, Figure S1: ANXA2 depleted cancer cells show enhanced AKT phosphorylation/activation upon EGF treatment compared to control cells, Figure S2: ANXA2 depleted cells show higher proliferation rate compared to matched control cells following EGF treatment, Figure S3: Analysis of ANXA2 shRNA cancer cell lines, Figure S4: Analysis of intracellular ROS levels in ANXA2 depleted versus control cells, Figure S5: Analysis of PRDX2 expression in ANXA2 KD cancer cells, Figure S6: HT1080 and MDA-MB-231 ANXA2 knockout cells show enhanced PRDX2 expression compared to control cells, Figure S7: PRDX2 depleted cancer cells show identical levels of ANXA2 expression compared to control cells, Figure S8: Immunofluorescence staining of ANXA2 and PRDX2 in colon clinical samples. Table S1: Analysis of ROS related genes in ANXA2 depleted versus control cancer cells, Table S2: List of shRNA and CRISPR/Cas9 plasmids, Table S3: Antibodies list, Table S4: qRT-PCR primers list.

## Appendix A

*Intracellular ROS Measurement*: 2 × 10^4^ cells/well were seeded in 96 wells plates and incubated overnight at 37 °C. Cell medium was removed and cells were incubated with serum free medium for 16 h (MDA-MB-231, A549) or 6 h (HT1080). Cells were then incubated with 50 μM DCF-DA (SIGMA) for 30 min at 37 °C, 5% CO_2_. After what fluorescence was measured (Excitation: 488 nm, Emission: 535 nm) using a fluorometer plate reader.

*MTS Assay*: 1 × 10^4^ cells/well were seeded in 96 wells plates and incubated overnight at 37 °C. After what cells were washed with PBS and incubated overnight (16 h) with serum free DMEM media. Cells were then either not treated or treated with 15 nM EGF for 24 h. Cell proliferation was determined using the CellTiter 96 AQueous Non-Radioactive Cell Proliferation Assay (Promega, Madison, WI, USA) according to the manufacturer’s instructions.

## References

[1] Madureira P.A., Hill R., Miller V.A., Wing P., Lee K., Waisman D.M. (2011). Annexin A2 is a novel Cellular Redox Regulatory Protein involved in Tumorigenesis. Oncotarget.

[2] Veal E.A., Day A.M., Morgan B.A. (2007). Hydrogen Peroxide Sensing and Signaling. Mol. Cell.

[3] Rhee S.G. (2006). H_2_O_2_, a necessary evil for cell signaling. Science.

[4] Madureira P.A., Waisman D.M. (2013). Annexin A2: The importance of being redox sensitive. Int. J. Mol. Sci..

[5] Acharya A., Das I., Chandhok D., Saha T. (2010). Redox regulation in cancer: A double-edged sword with therapeutic potential. Oxid. Med. Cell. Longev..

[6] Ray P.D., Huang B.W., Tsuji Y. (2012). Reactive oxygen species (ROS) homeostasis and redox regulation in cellular signaling. Cell. Signal..

[7] Carpenter G., Cohen S. (1990). Epidermal growth factor. J. Biol. Chem..

[8] Wiley H.S., Shvartsman S.Y., Lauffenburger D.A. (2003). Computational modeling of the EGF-receptor system: A paradigm for systems biology. Trends Cell Biol..

[9] Oda K., Matsuoka Y., Funahashi A., Kitano H. (2005). A comprehensive pathway map of epidermal growth factor receptor signaling. Mol. Syst. Biol..

[10] Liou G.-Y., Storz P. (2010). Reactive oxygen species in cancer. Free Radic. Res..

[11] Karnoub A.E., Weinberg R.A. (2008). Ras oncogenes: Split personalities. Nat. Rev. Mol. Cell Biol..

[12] Ward A.F., Braun B.S., Shannon K.M. (2012). Targeting oncogenic Ras signaling in hematologic malignancies. Blood.

[13] Scita G., Tenca P., Frittoli E., Tocchetti A., Innocenti M., Giardina G., Di Fiore P.P. (2000). Signaling from Ras to Rac and beyond: Not just a matter of GEFs. EMBO J..

[14] Castaldo S.A., Freitas J.R., Conchinha N.V., Madureira P.A. (2016). The Tumorigenic Roles of the Cellular REDOX Regulatory Systems. Oxid. Med. Cell. Longev..

[15] Madureira P.A., Hill R., Lee P.W.K., Waisman D.M. (2012). Genotoxic agents promote the nuclear accumulation of annexin A2: Role of annexin A2 in mitigating DNA damage. PLoS ONE.

[16] Madureira P.A., O’Connell P.A., Surette A.P., Miller V.A., Waisman D.M. (2012). The biochemistry and regulation of S100A10: A multifunctional plasminogen receptor involved in oncogenesis. J. Biomed. Biotechnol..

[17] Madureira P.A., Surette A.P., Phipps K.D., Taboski M.A.S., Miller V.A., Waisman D.M. (2011). The role of the annexin A2 heterotetramer in vascular fibrinolysis. Blood.

[18] Leslie N.R., Bennett D., Lindsay Y.E., Stewart H., Gray A., Downes C.P. (2003). Redox regulation of PI 3-kinase signalling via inactivation of PTEN. EMBO J..

[19] Arrigo A.P., Darlix J.L., Spahr P.F. (1983). A cellular protein phosphorylated by the avian sarcoma virus transforming gene product is associated with ribonucleoprotein particles. EMBO J..

[20] Rhee S.G., Woo H.A. (2011). Multiple functions of peroxiredoxins: Peroxidases, sensors and regulators of the intracellular messenger H_2_O_2_, and protein chaperones. Antioxid. Redox Signal..

[21] Gerke V., Moss S.E. (2002). Annexins: From structure to function. Physiol. Rev..

[22] Plishker G.A., Chevalier D., Seinsoth L., Moore R.B. (1992). Calcium-activated potassium transport and high molecular weight forms of calpromotin. J. Biol. Chem..

[23] Babiychuk E.B., Draeger A. (2000). Annexins in cell membrane dynamics. Ca(2+)-regulated association of lipid microdomains. J. Cell Biol..

[24] Moore R.B., Mankad M.V., Shriver S.K., Mankad V.N., Plishker G.A. (1991). Reconstitution of Ca(2+)-dependent K+ transport in erythrocyte membrane vesicles requires a cytoplasmic protein. J. Biol. Chem..

[25] Moore R.B., Plishker G.A., Shriver S.K. (1990). Purification and measurement of calpromotin, the cytoplasmic protein which activates calcium-dependent potassium transport. Biochem. Biophys. Res. Commun..

[26] Lee K.W., Lee D.J., Lee J.Y., Kang D.H., Kwon J., Kang S.W. (2011). Peroxiredoxin II restrains DNA damage-induced death in cancer cells by positively regulating JNK-dependent DNA repair. J. Biol. Chem..

[27] Cao J., Schulte J., Knight A., Leslie N.R., Zagozdzon A., Bronson R., Manevich Y., Beeson C., Neumann C.A. (2009). Prdx1 inhibits tumorigenesis via regulating PTEN/AKT activity. EMBO J..

[28] Wood Z.A., Poole L.B., Karplus P.A. (2003). Peroxiredoxin evolution and the regulation of hydrogen peroxide signaling. Science.

[29] Jaganjac M., Matijevic T., Cindric M., Cipak A., Mrakovcic L., Gubisch W., Zarkovic N. (2010). Induction of CMV-1 promoter by 4-hydroxy-2-nonenal in human embryonic kidney cells. Acta Biochim. Pol..

[30] Livak K.J., Schmittgen T.D. (2001). Analysis of relative gene expression data using real-time quantitative PCR and the 2(-Delta Delta C(T)) Method. Methods.

