# Supplementary material for: Annexin A2 Regulates AKT Upon H2O2-Dependent Signaling Activation in Cancer Cells
Source: Cancers (Basel). 2019 Apr 7;11(4):492. doi: 10.3390/cancers11040492 (PMC6520733; doi:10.3390/cancers11040492)
Supplement: Supplementary file 1 [file cancers-11-00492-s001.pdf]

cancers

Supplementary Materials: Annexin A2 Regulates AKT upon H_2O_2-Dependent Signaling Activation in Cancer Cells

Stéphanie Anais Castaldo, Tom Ajime, Gisela Serrão, Fábio Anastácio, Joana Teixeira Rosa, Carman Anthony Giacomantonio, Alison Howarth, Richard Hill and Patrícia Alexandra Madureira

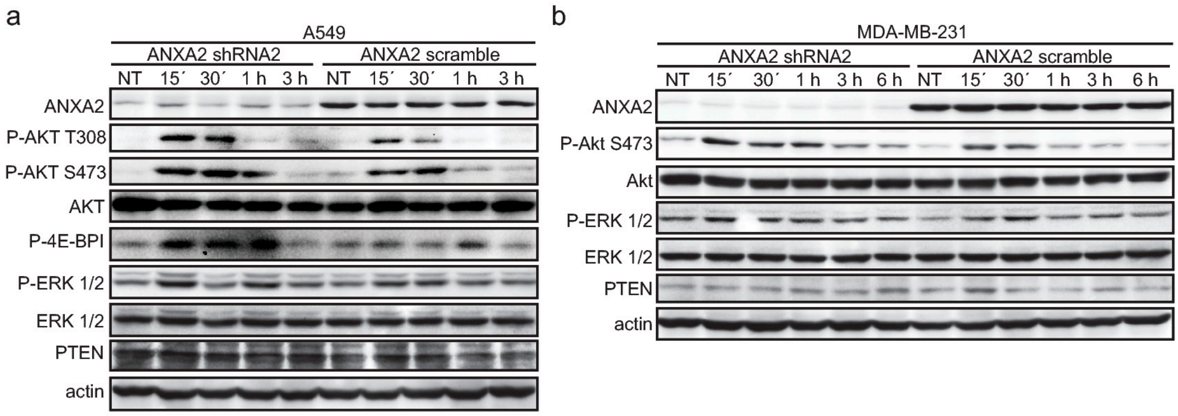

Figure S1. ANXA2 depleted cancer cells show enhanced AKT phosphorylation/activation upon EGF treatment compared to control cells. (**a**) A549, or (**b**) MDA-MB-231 cancer cells depleted of ANXA2 (ANXA2 shRNA1) or controls (ANXA2 scramble) were serum starved for 16 h and either not treated (NT) or treated with 15 nM EGF for the times indicated. After what cells were lysed and 20 µg of each protein extract was subjected to SDS-PAGE, transferred onto nitrocellulose membranes and analyzed by western blotting with the antibodies indicated. Results are representative of three independent experiments ($N = 3$).

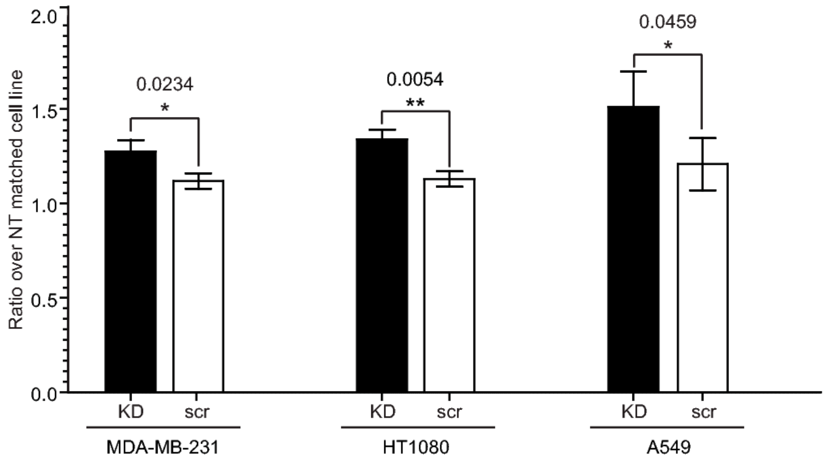

Figure S2. ANXA2 depleted cells show higher proliferation rate compared to matched control cells following EGF treatment. MDA-MB-231 ANXA2 shRNA2 (KD) and scramble (scr); HT1080 ANXA2 shRNA2 (KD) and scramble (scr); or A549 ANXA2 shRNA2 (KD) and scramble (scr) cells were serum starved for 16 h, after what they were either not treated (NT) or treated with 15 nM of EGF for 24 h. Cell proliferation was determined by using the CellTiter 96 AQueous Non-Radioactive Cell Proliferation Assay (Promega) according to the manufacturer's instructions. Error bars represent the standard deviation obtained from three independent experiments. Statistical analysis was evaluated

using two-tailed Student's *t*-test. In every case a *p*-value of less than 0.05 (*), less than 0.01(**) and 0.001 (***) was considered statistically significant.

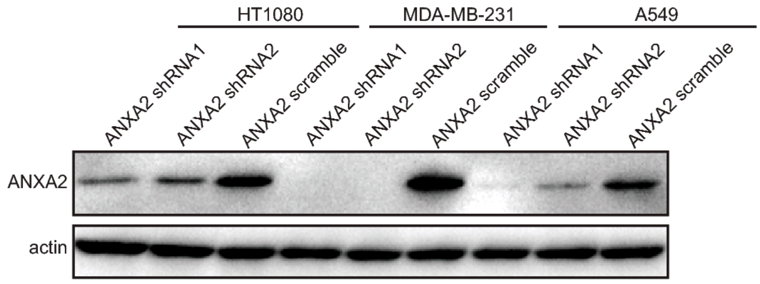

Figure S3. Analysis of ANXA2 shRNAs cancer cell lines. HT1080 (left lanes); MDA-MB-231 (middle lanes) or A549 (right lanes) ANXA2 shRNA1; ANXA2 shRNA2 or ANXA2 scramble cell lines were plated in 60 mm plates for 48 h. After what cells were lysed and 20 µg of each protein extract was subjected to SDS-PAGE, transferred onto nitrocellulose membranes and analyzed by western blotting with the antibodies indicated. Results are representative of three independent experiments (*N* = 3).

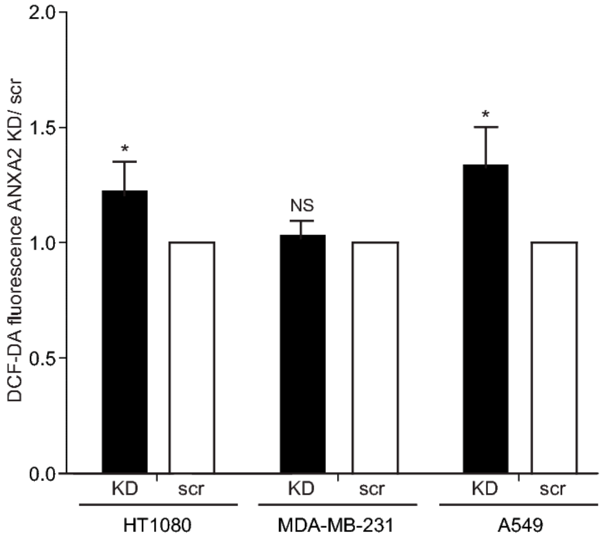

Figure S4. Analysis of intracellular ROS levels in ANXA2 depleted versus control cells. HT1080 ANXA2 shRNA2 (KD) and scramble (scr); MDA-MB-231 ANXA2 shRNA2 (KD) and scramble (scr); or A549 ANXA2 shRNA2 (KD) and scramble (scr) cells were incubated with 50 µM of DCF-DA for 30 min at 37 °C, 5% CO2. After what fluorescence was measured (Excitation: 488 nm, Emission: 535 nm) using a fluorometer plate reader. Error bars represent the standard deviation obtained from three independent experiments. Statistical analysis was evaluated using two-tailed Student's t-test. In every case a *p*-value of less than 0.05 (*), less than 0.01 (**) and 0.001 (***) was considered statistically significant.

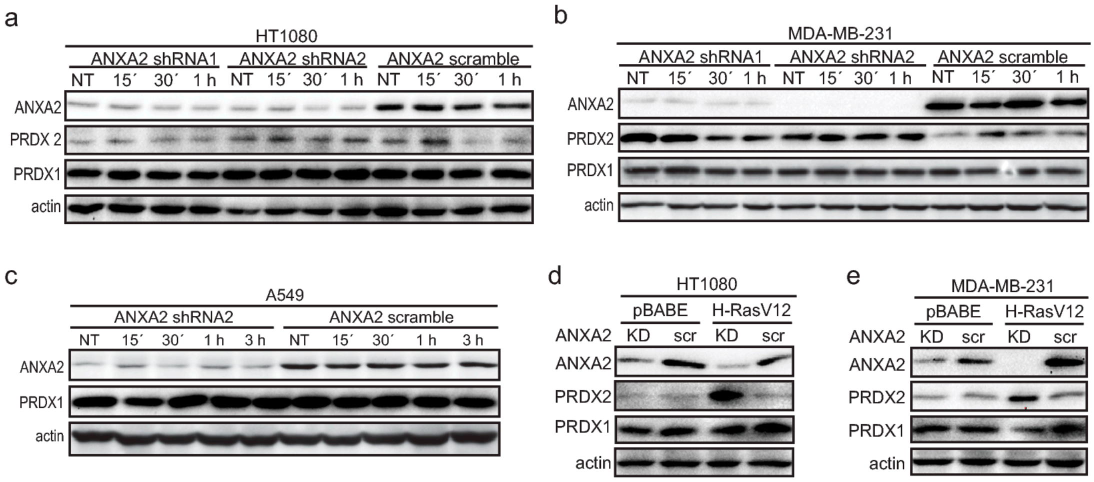

Figure S5. Analysis of PRDX2 expression in ANXA2 KD cancer cells. (**a**) HT1080, (**b**) MDA-MB-231, (**c**) A549 cancer cells depleted of ANXA2 (ANXA2 shRNA1, ANXA2 shRNA2) or controls (ANXA2 scramble) were serum starved for 6 h (HT1080) or for 16 h (MDA-MB-231) and either not treated (NT) or treated with 15 nM EGF for the times indicated; (**d**) HT1080 or (**e**) MDA-MB-231 ANXA2 knockdown (KD) and control (scr) cells expressing H-RasV12 or the empty vector pBABE were grown in serum free media for 6 h or 16 h, respectively. After what cells were lysed and 20 µg of each protein extract was subjected to SDS-PAGE, transferred onto nitrocellulose membranes and analyzed by western blotting with the antibodies indicated. Results are representative of at least three independent experiments.

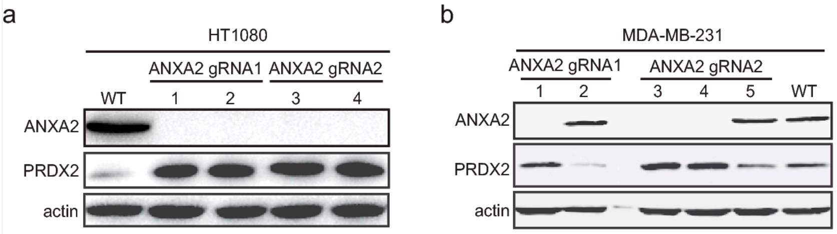

Figure S6. HT1080 and MDA-MB-231 ANXA2 knockout cells show enhanced PRDX2 expression compared to control cells. (**a**) HT1080 or (**b**) MDA-MB-231 cells were transfected with p36 gRNA-pX459 V2.0 CRIPR/Cas9 plasmids, selected with puromycin and serial dilutions were performed for selection of sub-populations. After what cells were lysed and 20 µg of each protein extract was subjected to SDS-PAGE, transferred onto nitrocellulose membranes and analyzed by western blot with the antibodies indicated.

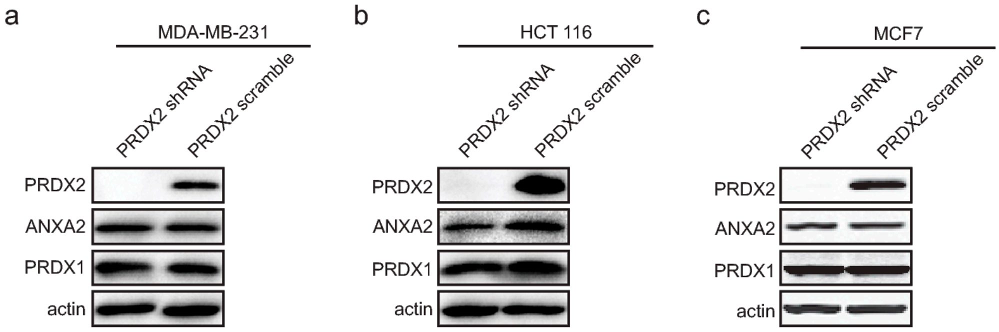

Figure S7. PRDX2 depleted cancer cells show identical levels of ANXA2 expression compared to control cells. (**a**) MDA-MB-23, (**b**) HCT 116, or (**c**) MCF7 cancer cells depleted of PRDX2 (PRDX2 shRNA1) or controls (PRDX2 scramble) were plated in a 60 mm plate containing DMEM complete media for 48 h. After what cells were lysed and 20 µg of each protein extract was subjected to SDS-

PAGE, transferred onto nitrocellulose membranes and analyzed by western blotting with the antibodies indicated.

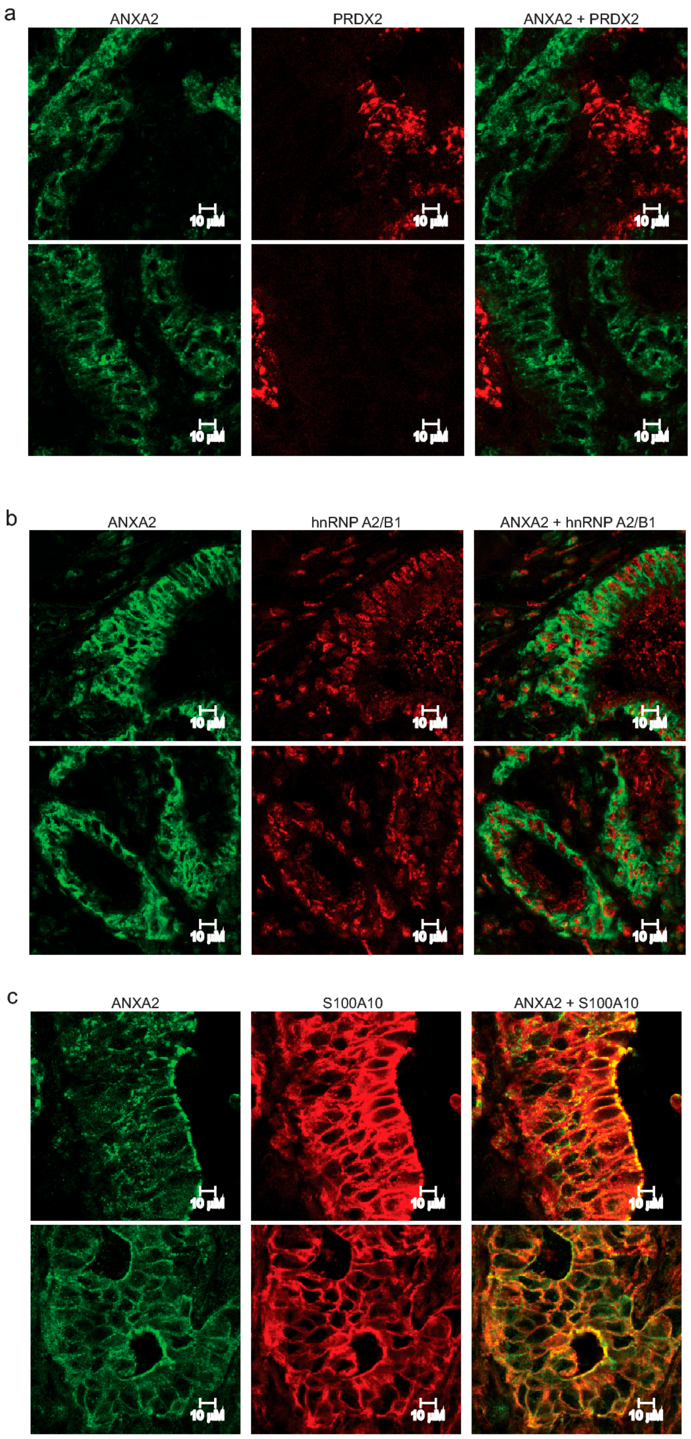

Figure S8. Immunofluorescence staining of ANXA2 and PRDX2 in colon clinical samples. Tumor clinical samples were flash frozen and sectioned using a cryostat. Samples were then fixed with methanol and immuno-stained with the antibodies indicated, followed by immunofluorescence staining with secondary antibodies (ANXA2-green; PRDX2-red). (**a**) ANXA2 and PRDX2 staining; (**b**) ANXA2 and hnRNP A2/B1 (nuclear marker) staining; (**c**) ANXA2 and S100A10 staining.

Table S1. Analysis of ROS related genes in ANXA2 depleted versus control cancer cells. HT1080 ANXA2 KO #1; ANXA2 KO #2 or WT or MDA-MB-231 ANXA2 shRNA1; ANXA2 shRNA2 or ANXA2 scramble cells were plated in 100 mm plates for 48 h. After what RNA extraction was performed using the RNeasy mini kit (QIAGEN) according to the manufacturer's instructions. A panel of 86 ROS dependent genes was analysed using the RT² Profiler™ PCR Array Human Oxidative Stress (Qiagen) according to the manufacturer's instructions in a LightCycler 96 instrument (Roche). Results are expressed as fold induction compared to controls (HT1080 WT and MDA-MB-231 ANXA2 scramble, respectively).

GENE	HT1080		MDA MB 231	
	ANXA2 KO #1	ANXA2 KO #2	ANXA2 shRNA1	ANXA2 shRNA2
ALB	1.6596	3.5991	2.6579	0.5932
ALOX12	0.5908	0.3836	0.8952	1.6319
AOX1	0.7427	0.6819	0.8952	1.0186
APOE	0.5908	1.7996	0.7902	0.6015
ATOX1	1.3955	1.4119	1.0212	0.9244
BNIP3	0.8832	0.8752	0.6463	0.5932
CAT	1.4751	2.0106	0.5626	0.7303
CCL5	7.4685	5.1253	0.7476	0.0876
CCS	0.785	1.7871	1.0945	1.5546
CYBB	0.5908	0.3836	0.6072	0.7252
CYGB	2.2358	2.9437	0.9661	0.26
DHCR24	1.0798	1.5558	0.4034	0.6814
DUOX1	0.5908	0.3836	0.2003	0.1463
DUOX2	0.5908	0.3836	0.7847	0.6184
DUSP1	1.6481	0.0113	0.889	1.3347
EPHX2	0.1936	0.1257	0.7847	0.6184
EPX	0.5908	0.4376	0.525	0.7303
FOXM1	0.9868	0.9984	1.0499	1.3255
FTH1	1.7664	1.5558	0.7793	0.7613
GCLC	0.7478	0.4957	0.9728	0.9636
GCLM	1.2149	0.9846	0.9462	0.7881
GPX1	1.249	1.6219	0.8952	1.4108
GPX2	0.865	1.1548	0.5286	2.2138
GPX3	0.5908	0.4346	0.603	0.6227
GPX4	1.1414	1.085	0.8587	0.8745
GPX5	0.5908	0.3836	0.7847	0.6184
GPX6	0.5908	0.3836	0.7847	0.6184
GPX7	0.5908	0.3836	0.7793	0.3968
GSR	0.6601	0.8166	0.5511	2.0091
GSS	0.9466	1.345	1.395	2.3239
GSTP1	0.8591	1.3544	1.1894	1.5439
GSTZ1	0.8472	2.7088	1.4951	2.1533
GTF2I	0.9937	1.2291	0.8528	0.9703
HMOX1	0.8241	1.3544	0.4602	1.1459
HSPA1A	0.5143	1.1078	0.8647	1.2453
KRT1	0.5908	0.3836	0.7847	0.6184
LPO	0.5908	0.3836	0.7847	0.6184
MB	0.5362	0.4468	1.3289	0.5651
MBL2	0.5908	0.3836	0.7847	0.6184
MGST3	1.0006	1.255	0.9661	0.9839
MPO	0.5908	0.3836	0.7847	0.6184
MPV17	1.2149	1.5666	0.9728	1.4206
MSRA	0.5868	1.4617	0.7476	1.7858
MT3	0.5908	0.3836	0.7847	0.7936
NCF1	3.8127	1.4924	0.457	0.8991
NCF2	0.2873	0.4316	0.4476	0.4988
NOS2	0.5908	0.3836	0.7847	0.6184
NOX4	0.871	0.7011	0.5397	0.918
NOX5	0.5908	0.3836	3.2722	1.7012
NQO1	1.3763	1.3829	1.0646	0.756

Table S1. *Cont.*

NUDT1	1.0006	1.8247	1.395	2.4226
OXR1	1.3574	1.0553	0.7322	0.8745
OXSR1	0.9598	0.6587	0.8012	0.7456
PDLIM1	0.5707	0.4755	1.0283	1.3347
PNKP	1.2577	2.3257	0.8181	1.5546
PRDX1	1.1179	0.8223	1.2572	1.1069
PRDX2	1.7787	1.6107	0.9864	1.344
PRDX3	0.9665	0.5774	0.6975	0.6314
PRDX4	0.9532	0.7833	0.9204	1.04
PRDX5	0.7905	1.3544	0.8352	1.0841
PRDX6	0.8356	1.0408	1.0572	1.2982
PREX1	2.5683	1.889	0.7847	0.6184
PRNP	0.7796	1.4821	0.9796	1.0766
PTGS1	0.5908	0.3836	0.8352	2.3892
PTGS2	0.2854	0.2887	1.0355	0.7991
PXDN	0.1191	0.1014	1.4047	1.6319
RNF7	0.6929	1.3733	1.1253	1.4913
SCARA3	1.0287	1.5238	0.2366	0.1172
VIMP	1.1102	1.4316	0.7072	0.957
SEPP1	0.6202	0.3836	1.1731	1.3072
SFTPD	0.5908	0.3836	0.7847	0.6184
SIRT2	0.9868	1.6331	0.7271	1.1302
SOD1	1.0145	0.6587	1.0499	0.6314
SOD2	0.9271	0.6772	0.8647	0.7827
SOD3	0.5908	0.3836	0.4538	0.4719
SQSTM1	1.8287	2.2002	0.5323	0.8867
SRXN1	1.1899	2.4244	0.9268	1.749
STK25	1.3859	1.9287	0.9268	1.2982
TPO	0.5908	0.3836	0.7847	0.6184
TTN	0.5908	1.1955	0.7847	0.6184
TXN	1.1025	0.7833	1.0427	0.7202
TXNRD1	1.4854	1.5132	0.7793	1.3347
TXNRD2	1.2404	1.9154	0.8528	1.1146
UCP2	0.5908	0.3836	0.5397	0.5891

Table S2. List of shRNA and CRISPR/Cas9 plasmids. Plasmids were constructed by cloning the following double stranded oligos into the backbone plasmids indicated.

Plasmid Name	5′-oligo	3′-oligo	Plasmid Backbone
pSUPER-retro-puro-ANXA2 shRNA1	5′-GAT CCC CCC TGG TTC AGT GCA TTC AGT TCA AGA GAC TGA ATG CAC TGA ACC AGG TTT TTA-3′	5′-AGC TTA AAA ACC TGG TTC AGT GCA TTC AGT CTC TTG AAC TGA ATG CAC TGA ACC AGG GGG-3′	pSUPER-retro-puro (Oligoengine)
pSUPER-retro-puro-ANXA2 shRNA2	5′-GAT CCC CGT GCA TAT GGG TCT GTC AAT TCA AGA GAT TGA CAG ACC CAT ATG CAC TTT TTA-3′	5′-AGC TTA AAA AGT GCA TAT GGG TCT GTC AAT CTC TTG AAT TGA CAG ACC CAT ATG CAC GGG-3′	pSUPER-retro-puro (Oligoengine)
pSUPER-retro-puro-ANXA2 scramble	5′-GAT CCC CGT GCA TAT GGG TCT GTC CAT TAG AGA GAT TGA CAG ACC CAT ATG CAC TTT TTA-3′	5′-AGC TTA AAA AGT GCA TAT GGG TCT GTC AAT CTC TCT AAT GGA CAG ACC CAT ATG CAC GGG-3′	pSUPER-retro-puro (Oligoengine)
pSUPER-retro-neo-ANXA2 shRNA1	5′-GAT CCC CCC TGG TTC AGT GCA TTC AGT TCA AGA GAC TGA ATG CAC TGA ACC AGG TTT TTA-3′	5′-AGC TTA AAA ACC TGG TTC AGT GCA TTC AGT CTC TTG AAC TGA ATG CAC TGA ACC AGG GGG-3′	pSUPER-neo-puro (Oligoengine)
pSUPER-retro-neo-ANXA2 shRNA2	5′-GAT CCC CGT GCA TAT GGG TCT GTC AAT TCA AGA GAT TGA CAG ACC CAT ATG CAC TTT TTA-3′	5′-AGC TTA AAA AGT GCA TAT GGG TCT GTC AAT CTC TTG AAT TGA CAG ACC CAT ATG CAC GGG-3′	pSUPER-neo-puro (Oligoengine)
pSUPER-retro-neo-ANXA2 scramble	5′-GAT CCC CGT GCA TAT GGG TCT GTC CAT TAG AGA GAT TGA CAG ACC CAT ATG CAC TTT TTA-3′	5′-AGC TTA AAA AGT GCA TAT GGG TCT GTC AAT CTC TCT AAT GGA CAG ACC CAT ATG CAC GGG-3′	pSUPER-neo-puro (Oligoengine)
pANXA2-gRNA1-px459-V2	5′-CAC CGC TAC ACC CCC AAG TGC ATA T-3′	5′-AAA CAT ATG CAC TTG GGG GTG TAG C-3′	px459-V2 (Addgene: 62988)
pANXA2-gRNA2-px459-V2	5′-CAC CGC TCA GCA TCA AAG TTA GTA T-3′	5′-AAA CAT ACT AAC TTT GAT GCT GAG C-3′	px459-V2 (Addgene: 62988)

Table S3. Antibodies list.

Target Protein	Reference	Company	Procedure
ANXA2 (D1/274.5)		Produced In house	Western blotting
PTEN (A2B1)	sc-7974	Santa Cruz Biotechnology (SCBT)	Western blotting
PRDX1	sc-7381	SCBT	Western blotting
PRDX2 (N-13)	sc-23967	SCBT	Western blotting; Immunofluorescence-IF
P-AKT Ser 473	sc-7985-R	SCBT	Western blotting
P-AKT Thr 308	9275	Cell Signaling Technology (CST)	Western blotting
AKT	9272	CST	Western blotting
P-ERK 1/2	sc-16982	SCBT	Western blotting
ERK 1/2 (MK1)	sc-135900	SCBT	Western blotting
H-Ras	sc-520	SCBT	Western blotting
GAPDH	sc-25778	SCBT	Western blotting
β-tubulin	sc-5286	SCBT	Western blotting
actin (C-11)	sc-1615	SCBT	Western blotting
p-4EBPI (62.Ser 65)	sc-293124	SCBT	Western blotting
SQSTM1 (D-3)	sc-28359	SCBT	Western blotting
MTH1 (H-1)	sc-271082	SCBT	Western blotting
catalase (H-9)	sc-271803	SCBT	Western blotting
SOD-1 (G-11)	sc-17767	SCBT	Western blotting
Trx (A-5)	sc-166393	SCBT	Western blotting
GSR (C-10)	sc-133245	SCBT	Western blotting
GPX-3	sc-58361	SCBT	Western blotting
TrxRD (B-2)	sc-28321	SCBT	Western blotting
GFP (FL)	sc-8334	SCBT	Immunoprecipitation (IP)
Rabbit IgG	NI01-100UG	Merk	IP
ANXA2	ab41803-100	AbCam	IF
S100A10 (4E7E10)	sc-81153	SCBT	IF
hnRNP A2/B1 (B-7)	sc-374053	SCBT	IF
anti-mouse 488 Alexa Fluor	A-11001	Molecular Probes	IF
anti-rabbit 488 Alexa Fluor	A-11008	Molecular Probes	IF
anti-goat 488 Alexa Fluor	A-11055	Molecular Probes	IF
anti-mouse 546 Alexa Fluor	A-11003	Molecular Probes	IF

Table S4. qRT-PCR primers list.

Gene	Forward Primer	Reverse Primer
PRDX2	5′-GTC CTT CGC CAG ATC ACT GT-3′	5′-ACG TTG GGC TTA ATC GTG TC-3′
EPHX2	5′-GTG TTC ATT GGC CAT GAC TG-3′	5′-CTC AGT GAC CAT CCT GCT GA-3′
SrxN1	5′-GGG CTG GAC CCA CTG TAG TA-3′	5′-CCA GGG ACT CTT GGT TTT CA-3′
NUDT1	5′-AAG AAG GAG AGA CCA TCG AGG AT-3′	5′-TGC AGG GCG TCC ACT GT-3′
CATALASE	5′-TGA CCG AGA GAG AAT TCC TGA-3′	5′-CCT TTG CCT TGG AGT ATT TGG-3′
SCARA-3	5′-TGC GGA TTC TTT ACC TCT TCC T-3′	5′-TCT TCG GAG AGA GAG TCC ACT TTT-3′
TxnRD1	5′-TTC ACC TCA GTT TTC TTC ACT CC-3′	5′-TCT GCC CTC CTG ATA AGC AA-3′
TxnRD2	5′-GGT GGA CTA CGT GGA ACC TT-3′	5′-TCT GCC ATC TTC CTC CAG TCA-3′
RPLP0	5′-AGA CAA TGT GGG CTC CAA GCA GAT-3′	5′-GCA TCA TGG TGT TCT TGC CCA TCA-3′

© 2019 by the authors. Submitted for possible open access publication under the terms and conditions of the Creative Commons Attribution (CC BY) license (http://creativecommons.org/licenses/by/4.0/).
